# The Influence of Analysis Mode Selection on Prediction Accuracy of Corneal Astigmatism Using Pentacam

**DOI:** 10.3389/fmed.2021.713502

**Published:** 2021-10-15

**Authors:** Jiaqi Meng, Jifeng Yu, Wenwen He, Ling Wei, Yi Lu, Xiangjia Zhu

**Affiliations:** ^1^Eye Institute, Eye & Ear, Nose and Throat (ENT) Hospital of Fudan University, Shanghai, China; ^2^Department of Ophthalmology, National Center for Children's Health, Beijing Children's Hospital, Capital Medical University, Beijing, China; ^3^Key Laboratory of Myopia, Ministry of Health, Shanghai, China; ^4^Key Laboratory of Visual Impairment and Restoration, Shanghai, China; ^5^National Health Commission (NHC) Key Laboratory of Myopia (Fudan University), Shanghai, China; ^6^Laboratory of Myopia, Chinese Academy of Medical Sciences, Shanghai, China

**Keywords:** corneal astigmatism, Pentacam, toric intraocular lens, cataract, ocular biometry

## Abstract

**Purpose:** To evaluate the influence of analysis mode selection on prediction accuracy of corneal astigmatism using Pentacam.

**Methods:** Fifty-nine eyes of 59 patients implanted with toric intraocular lenses (IOLs) were included in the retrospective study. Preoperative corneal astigmatism (total refractive power) measured with Pentacam was analyzed based on 2-, 3-, 4-, or 5-mm ring or zone mode either centered on corneal apex or pupil center. Actual corneal astigmatism was calculated based on residual astigmatism on the corneal plane, surgical-induced astigmatism, and effective toric power on the corneal plane. Prediction error, the difference between actual corneal astigmatism and measured astigmatism, was compared among different analysis modes. Influences of local topography on prediction error were also evaluated.

**Results:** Based on the zone mode, prediction error was lower when centered on corneal apex than on pupil center at different diameters, whereas based on the ring mode, this difference was only seen at 2-mm cornea (all *P* < 0.05). When centered on the corneal apex, the zone mode showed lower prediction error than the ring mode at 4- and 5-mm corneas (both *P* < 0.001), regardless of asymmetric or symmetric astigmatism. In symmetric bowtie, the zone mode showed lower prediction error than the ring mode at 2-mm cornea of the small bowtie, and 4- and 5-mm corneas of the large bowtie (all *P* < 0.05).

**Conclusions:** For toric IOL planning, the corneal apex may be a better reference center. At a cornea diameter ≥4 mm, the zone mode is more accurate than the ring mode. Local topography affects prediction accuracy in the symmetric bowtie.

## Introduction

With the increasing visual demands of patients, cataract surgery has developed into a refractive procedure in recent years, not only aiming to remove the opacified lens but also to achieve better spectacle-free visual outcomes (1). However, preoperative astigmatism of 1.25 D or greater is present in 20–30% of cataract patients (2, 3), which may lead to post-operative blurred vision and reduced visual quality, if not corrected during surgery (4, 5). With improvements in intraocular lens (IOL) design, toric IOLs are now widely used to correct preexisting corneal astigmatism (6, 7).

For toric IOL surgical planning, Pentacam is a very useful facility (8). Based on the Scheimpflug rotating camera, it provides more details of the cornea and thus improves the accuracy of toric IOL planning (9). The instrument has several analytical modes, yielding multiple sets of data for corneal diameters ranging from 1.0 to 8.0 mm, based on a ring or zone mode, and centered either on the corneal apex or the pupil center. With the ring mode, corneal astigmatism is calculated with data points locating on the ring, while with the zone mode, all the keratometric data points within the diameter are included in the analysis (10). However, it remained unclear which analysis mode is more accurate for the toric IOL surgical planning. Moreover, the influences of the local corneal topography on prediction accuracy are still unclear, as even in eyes with symmetric bowtie of corneal astigmatism, the location of the steepest part of the bowtie varies.

Thus, in this study, we firstly compared the prediction error for corneal astigmatism based on 2-, 3-, 4-, or 5-mm ring or zone mode either centered on the corneal apex or the pupil center and then analyzed the influence of the local topography on the prediction error in eyes with symmetric bowtie of corneal astigmatism.

## Materials and Methods

This retrospective study adhered to the principles of the Declaration of Helsinki. The protocols of the study were approved by the institutional review board of the Eye and Ear, Nose, and Throat (EENT) Hospital of Fudan University, Shanghai, China. Written informed consent was routinely obtained from each participant before surgery for the use of their clinical data.

### Patient Selection

Medical records of patients undergoing cataract surgery at the EENT Hospital of Fudan University between October 1, 2018, and November 1, 2019, were reviewed. Eyes with uneventful cataract surgery and toric IOL implantation (AT Torbi 709M IOL, Carl Zeiss AG, Oberkochen, Germany) were included. Exclusion criteria were: eyes with poor quality of Pentacam scans, corneal pathologies such as keratoconus and corneal scars, contact lens wearing within 2 weeks before examinations, previous intraocular surgery or trauma, zonular weakness, glaucoma, severe fundus pathology, uveitis, and systemic disorders such as diabetes. Finally, 59 eyes of 59 patients were available for analysis.

### Preoperative Examinations

All patients underwent complete ophthalmic examinations before surgery, namely, visual acuity testing, slit-lamp examination, corneal topography (Pentacam HR, Oculus Inc., Wetzlar, Germany), biometry measurements (IOLMaster700, Carl Zeiss AG, Oberkochen, Germany), fundoscopy, and B-scan ultrasonography. The cylinder power and axis of toric IOLs were calculated based on the corneal refractive power (axial/sagittal front) using the online calculator of the IOL manufacturer (ZCALC2: https://zcalc.meditec.zeiss.com).

### Surgical Procedure

All surgeries were performed with a standard procedure by one experienced surgeon (Prof. YL). A 2.6-mm clear corneal incision was made temporally and then a 5.5-mm continuous curvilinear capsulorhexis was created, followed by hydrodissection, phacoemulsification, and cortex removal. Using the Callisto Eye System (Carl Zeiss AG, Oberkochen, Germany), the toric IOL was implanted in the capsular bag. After the thorough removal of the viscoelastic from above and below the IOL, the alignment was checked again and the incision was hydrated. At the end of the surgery, the axis of the toric IOL was recorded for each eye. All eyes received routine post-operative medications after surgery.

### Post-operative Examinations

At 1 month after surgery, the post-operative examinations included visual acuity testing, manifest refraction, corneal topography, and anterior segment photography. The uncorrected distance visual acuity (UDVA; logarithm of the minimal angle of resolution, logMAR) and the corrected distance visual acuity (CDVA; logMAR) were recorded for each eye. During the photography of the anterior segment, a retroilluminated photograph was taken after pupil dilation to measure the post-operative axis of the IOL at 1 month.

### Prediction Error Calculation

Preoperative corneal astigmatism (total refractive power) measured with Pentacam was analyzed based on the 2-, 3-, 4-, or 5-mm ring or zone mode centered either on the corneal apex or the pupil center, resulting in 16 sets of astigmatism data for each eye.

The actual corneal astigmatism was calculated as the vector sum of the post-operative residual astigmatism on the corneal plane (*D*_residual/cornea_), surgical-induced astigmatism (SIA), and the effective toric IOL power on the corneal plane (*D*_IOL/cornea_). Post-operative residual astigmatism (*D*_residual_) was converted to the corneal plane (*D*_residual/cornea_) with the formula (11):


(1) $$\begin{array}{l} D_{\text{residual}/\text{cornea}}=\;\frac{D_{\text{residual}}}{1-0.012\times D_{\text{residual}}} \end{array}$$

Surgical-induced astigmatism was calculated as the vector difference between post-operative and preoperative total corneal astigmatism both measured with Pentacam. The effective toric IOL power (*D*_IOL_) was also converted to the corneal plane (*D*_IOL/cornea_) by (12, 13):


(2) $$\begin{array}{l} D_{\text{IOL}/\text{cornea}}=\;\frac{1,336}{\frac{1,336}{\frac{1,336}{\frac{1,336}{K}-\text{ELP}}+D_{\text{IOL}}}\;+\text{ ELP}}-K \end{array}$$

where *K* is the net corneal power measured with Pentacam and ELP is the effective lens position calculated from the anterior chamber depth after surgery. In addition, the post-operative axis of the toric IOL at 1 month was used for the vector analysis.

The prediction error, defined as the vector difference between the actual corneal astigmatism and the preoperative corneal astigmatism measured with Pentacam, was compared among different analysis modes. Both the absolute prediction error and the centroid error were calculated in the study.

### Local Corneal Topography of the Bowtie Pattern

The total corneal irregular astigmatism was recorded with Pentacam for all eyes, and the values of <0.3, ≥0.3, and <0.5, and ≥0.5 μm were defined as mild, moderate, and severe irregularity, respectively. Based on the corneal topography, eyes with an inferior-superior index (I-S) of >1.5 D, a superior-inferior index (S-I) of >2.5 D, or a skewed radial axis index (SRAX) of >22° were classified as asymmetric, while all the other eyes were classified as symmetric (14). I-S was defined as the difference in corneal curvature between the inferior and superior points on the steep axis at 5 mm in the sagittal curvature (front) image, and S-I was defined as the difference between the superior and inferior points (14). SRAX was defined as the difference in the axes of the two lobes of the bowtie (14).

**Figure 1:**
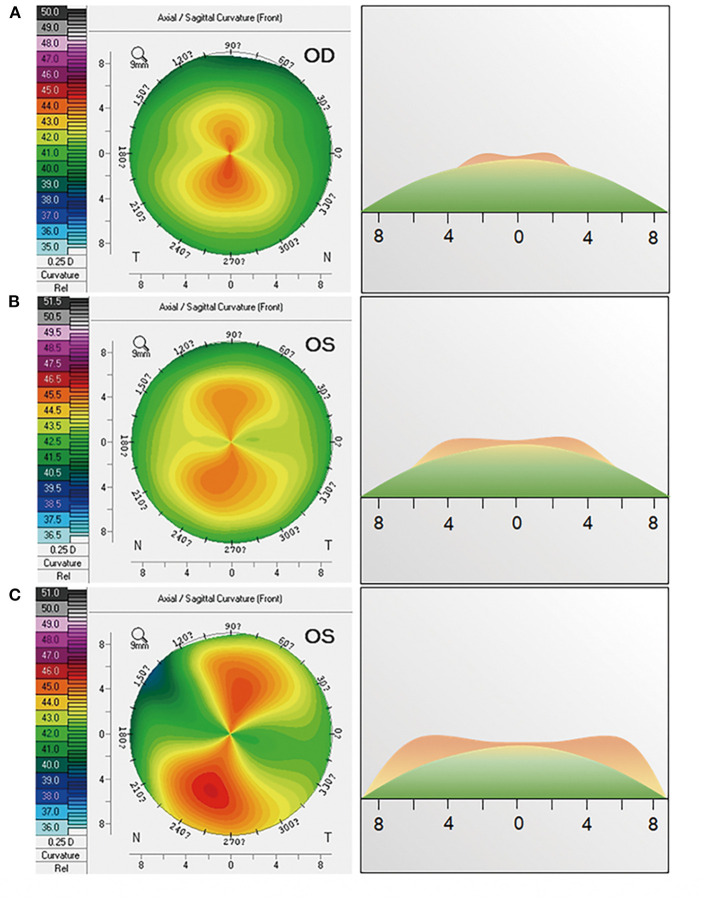
Influences of the local corneal topography on prediction error in a symmetric bowtie. The first column shows the corneal topography of **(A)** eyes with small bowtie (type A, diameter ≤5mm), **(B)** eyes with medium bowtie (type B, 5 mm < diameter ≤7 mm), and **(C)** eyes with large bowtie (type C, diameter >7 mm). The second column shows the topographic profile on the steep axis corresponding to the first column.

In the symmetric group, the eyes were further divided into three subgroups according to the diameter of symmetric bowtie: type A (small bowtie, diameter ≤ 5 mm; Figure 1A); type B (medium bowtie, 5 mm < diameter ≤ 7 mm; Figure 1B); and type C (large bowtie, diameter >7 mm; Figure 1C). To measure the diameter of the bowtie, a color scale with increments of 0.25 D was used.

### Statistical Analysis

The sample size required to detect a difference of 0.20 D with an SD of 0.50 D was calculated as 54 eyes for a significance level of 5% and a test power of 0.80. Continuous data are presented as means ± SDs. Shapiro–Wilk tests were performed for Gaussian distributions. The paired Wilcoxon signed-rank test was used for the comparison of median absolute errors. A paired *t*-test was used to compare mean absolute errors. All statistical analyses were performed with SPSS version 22 (SPSS, Chicago, Illinois, USA). A *P* < 0.05 was considered statistically significant.

## Results

### Characteristics

Table 1 shows the characteristics of the included eyes. The mean age of the patients included was 63.4 ± 14.0 years. The mean axial length was 26.50 ± 2.81 mm. The mean preoperative total corneal refractive power measured with Pentacam was 2.09 ± 0.74 D. At 1 month after surgery, the mean residual astigmatism was 0.62 ± 0.41 D.

**Table 1:** Characteristics.

	**Total (*N* = 59)**
Age (years)	63.44 ± 14.00
Sex (male/female)	23/36
Eye laterality (right/left)	32/27
UCVA (logMAR)	0.24 ± 0.13
CDVA (logMAR)	0.09 ± 0.11
AL (mm)	26.50 ± 2.81
Anterior chamber depth (mm)	3.18 ± 0.46
Pupil diameter (mm)	2.69 ± 0.55
Preoperative TCRP^*^ (D)	2.09 ± 0.74
IOL cylinder power (D)	2.39 ± 1.00
SIA (D)	0.55 ± 0.38
Residual astigmatism (D)	0.62 ± 0.41

*N, number; UCVA, uncorrected visual acuity; CDVA, corrected distance visual acuity; AL, axial length; TCRP, total corneal refractive power; D, diopters; IOL, intraocular lens; SIA, surgical induced astigmatism*.

*Data are mean ± SD*.

* *Measured with Pentacam*.

### Prediction Errors of Corneal Astigmatism Based on Different Analysis Modes

Table 2 shows the prediction errors of corneal astigmatism based on the ring or zone mode centered on either the corneal apex or pupil center. For the zone mode, the absolute prediction errors of corneal astigmatism were significantly lower when centered on the corneal apex than centered on the pupil center at 2-, 3-, 4-, and 5-mm corneas (paired Wilcoxon signed-rank test, all *P* < 0.05); whereas, for the ring mode, this difference was only seen for a corneal diameter of 2 mm (paired Wilcoxon signed-rank test, *P* = 0.01).

**Table 2:** Prediction errors of corneal astigmatism based on the ring or zone mode either centered on the corneal apex or pupil center.

	**Corneal apex**		**Pupil center**		***P* value**
	**Mean ± SD**	**Median (interquartile range)**	**Mean ± SD**	**Median (interquartile range)**	
**Absolute prediction error^†^ (D)**					
**Ring**					
2 mm	0.57 ± 0.45	0.47 (0.20–0.90)	0.64 ± 0.52	0.47 (0.22–1.00)^*^	0.010^*^
3 mm	0.60 ± 0.54	0.43 (0.20–0.88)	0.64 ± 0.55	0.46 (0.18–0.92)	0.137
4 mm	0.74 ± 0.54	0.67 (0.31–1.00)^*^	0.74 ± 0.56	0.58 (0.31–1.15)^*^	0.471
5 mm	0.88 ± 0.62	0.81 (0.36–1.18)^*^	0.88 ± 0.63	0.73 (0.32–1.25)^*^	0.949
**Zone**					
2 mm	0.62 ± 0.45	0.55 (0.29–0.97)	0.83 ± 0.61	0.67 (0.38–1.29)^*^	<0.001^*^
3 mm	0.58 ± 0.47	0.43 (0.22–0.82)	0.69 ± 0.54	0.47 (0.28–1.10)	<0.001^*^
4 mm	0.61 ± 0.49	0.45 (0.22–0.88)^*^	0.66 ± 0.55	0.51 (0.24–1.01)^*^	0.010^*^
5 mm	0.67 ± 0.52	0.55 (0.27–0.92)^*^	0.72 ± 0.55	0.58 (0.30–1.11)^*^	0.005^*^
**Mean centroid error (D @ degree)**					
**Ring**					
2 mm	0.19 @ 148		0.21 @ 141		–
3 mm	0.25 @ 141		0.25 @ 140		–
4 mm	0.36 @ 137		0.35 @ 137		–
5 mm	0.44 @ 130		0.43 @ 129		–
**Zone**					
2 mm	0.28 @ 149		0.29 @ 130		–
3 mm	0.18 @ 150		0.23 @ 136		–
4 mm	0.21 @ 144		0.24 @ 137		–
5 mm	0.31 @ 139		0.32 @ 137		–

*D, diopters*.

* *P < 0.05 were considered statistically significant*.

† *Paired Wilcoxon signed-rank test*.

When centered on the corneal apex, the zone mode showed significantly lower absolute prediction error than the ring mode at 4- and 5-mm cornea (paired Wilcoxon signed-rank test, both *P* < 0.001). When centered on the pupil center, differences between ring and zone mode were seen at 2-, 4-, and 5-mm cornea (paired Wilcoxon signed-rank test, all *P* < 0.05). The zone mode also showed lower mean centroid error than the ring mode at 3-, 4-, and 5-mm corneas (0.18 vs. 0.25 D, respectively, for 3 mm, 0.21 vs. 0.36 D, respectively, for 4 mm, 0.31 vs. 0.44 D, respectively, for 5 mm). Based on the zone mode, the lowest median absolute error (0.43 D) and mean centroid error (0.18 D) were both seen at the 3-mm cornea.

All the included eyes had mild irregularity. Of the 59 eyes, 23 (39%) had asymmetric astigmatism. Table 3 shows the prediction errors of corneal astigmatism in the asymmetric and symmetric groups between the ring and zone modes when centered on the corneal apex. In both the groups, the zone mode showed significantly lower absolute prediction error than the ring mode at 4- and 5-mm cornea (paired Wilcoxon signed-rank test, all *P* < 0.05), while in the asymmetric group, the zone mode showed higher absolute prediction error than the ring mode at 2-mm cornea (paired Wilcoxon signed-rank test, *P* = 0.03).

**Table 3:** Prediction errors of corneal astigmatism based on the ring or zone mode centered on the corneal apex in the asymmetric and symmetric groups.

**Absolute prediction error (D)**	**Ring**		**Zone**		***P*-value^†^**
	**Mean ± SD**	**Median (interquartile range)**	**Mean ± SD**	**Median (interquartile range)**	
**Asymmetric group**					
2 mm	0.49 ± 0.41	0.33 (0.16–0.89)	0.59 ± 0.42	0.46 (0.33–0.99)	0.035^*^
3 mm	0.51 ± 0.50	0.43 (0.10–0.75)	0.50 ± 0.42	0.33 (0.17–0.79)	0.561
4 mm	0.66 ± 0.58	0.49 (0.27–0.98)	0.52 ± 0.42	0.43 (0.20–0.69)	0.019^*^
5 mm	0.84 ± 0.73	0.73 (0.32–1.18)	0.59 ± 0.53	0.49 (0.21–0.85)	0.001^*^
**Symmetric group**					
2 mm	0.62 ± 0.47	0.55 (0.26–0.91)	0.63 ± 0.48	0.56 (0.26–0.95)	0.787
3 mm	0.65 ± 0.57	0.48 (0.22–0.97)	0.64 ± 0.50	0.56 (0.24–0.87)	0.504
4 mm	0.79 ± 0.52	0.74 (0.38–1.08)	0.66 ± 0.53	0.59 (0.31–1.01)	<0.001^*^
5 mm	0.91 ± 0.54	0.83 (0.53–1.20)	0.72 ± 0.52	0.65 (0.30–0.99)	0.002^*^

*D, diopters*.

* *P < 0.05 were considered statistically significant*.

† *Paired Wilcoxon signed-rank test*.

### Influences of the Local Corneal Topography on Prediction Error in Symmetric Bowtie

Among the eyes with symmetric bowtie of corneal astigmatism, the proportions of type A, type B, and type C were 17% (6/36), 50% (18/36), and 33% (12/36), respectively. Table 4 shows the prediction errors of corneal astigmatism in the eyes with the symmetric bowtie pattern. In type A, the zone mode showed a significantly lower absolute prediction error than the ring mode at 2-mm cornea (paired *t*-test, *P* = 0.02). In type B, no differences were identified at any corneal diameters between the two modes (paired *t*-test, all *P* > 0.05). In type C, the zone mode had significantly lower absolute prediction errors than the ring mode at 4- and 5-mm cornea (paired *t*-test, *P* = 0.004 for 4 mm and *P* = 0.001 for 5 mm).

**Table 4:** Prediction errors of corneal astigmatism based on the ring or zone mode centered on the corneal apex in eyes with a symmetric bowtie.

**Absolute prediction error (D)**	**Ring**	**Zone**	***P*-value^†^**
**Type A**			
2 mm	0.92 ± 0.74	0.75 ± 0.69	0.020^*^
3 mm	1.01 ± 0.83	0.88 ± 0.70	0.221
4 mm	1.01 ± 0.71	0.92 ± 0.76	0.194
5 mm	1.04 ± 0.52	0.97 ± 0.70	0.348
**Type B**			
2 mm	0.59 ± 0.44	0.64 ± 0.50	0.231
3 mm	0.62 ± 0.56	0.63 ± 0.51	0.897
4 mm	0.76 ± 0.54	0.66 ± 0.52	0.071
5 mm	0.77 ± 0.60	0.71 ± 0.52	0.478
**Type C**			
2 mm	0.53 ± 0.33	0.56 ± 0.35	0.454
3 mm	0.53 ± 0.39	0.53 ± 0.35	0.983
4 mm	0.74 ± 0.38	0.53 ± 0.39	0.004^*^
5 mm	1.02 ± 0.45	0.62 ± 0.40	0.001^*^

*D, diopters*.

*Data are mean ± SD*.

* *P < 0.05 were considered statistically significant*.

† *Paired t-test*.

## Discussion

Over the past decade, the implantation of toric IOLs has become a preferred option for correcting preexisting corneal astigmatism and achieving better post-operative visual outcomes for cataract patients (15, 16). Pentacam is a useful instrument for planning toric IOL surgery and improving prediction accuracy for corneal astigmatism (17, 18). With various analysis modes, it provides more than 30 sets of keratometric data for corneal diameters ranging from 1.0 to 8.0 mm, based on the ring or zone mode centered on either the corneal apex or pupil center (10). However, it remained unclear which analysis mode is most appropriate for the toric IOL surgical planning. Moreover, little was known about the influences of the local corneal topography on prediction error. In this study, we evaluated the prediction errors among different analysis modes of Pentacam and found that for toric IOL surgical planning, the corneal apex may be a better reference center than the pupil center, and at a corneal diameter of ≥4 mm, the zone mode may be more accurate than the ring mode. Prediction accuracy may also be affected by the local topography of symmetric bowtie astigmatism.

The corneal apex is found as a better reference center for toric IOL surgical planning according to our data. Choosing the inappropriate reference center may reduce the prediction accuracy, especially when there is a certain distance between the two reference centers (19). Previously, some studies recommended the pupil center as the reference center in this context, because the researchers considered that the entrance pupil controls what the patient sees (20, 21). However, according to our study, the prediction accuracy of corneal astigmatism is higher when the measurement is centered on the corneal apex rather than on the pupil center, based on the 2-, 3-, 4-, or 5-mm zone mode or the 2-mm ring mode. The corneal apex usually corresponds to the center of the capsular bag, which is closely associated with the optical performance of the toric IOL (22), and this may explain why using the corneal apex as the reference center resulted in more accurate prediction in most cases. Another explanation may be that pupil-centered measurements made with Pentacam have poorer precision (23), given the dynamic nature of the pupil.

We also found that when centered on the corneal apex, the zone mode presented higher prediction accuracy than the ring mode at the corneal diameter of ≥4 mm, regardless of whether the astigmatism was asymmetric or symmetric. Unlike the ring mode in which corneal astigmatism is analyzed merely with data points over the ring, the zone mode includes all the data points within the corneal diameter for analysis (10). Thus, an assessment of corneal astigmatism based on the zone mode may be more representative of the actual corneal astigmatism in the selected area, and will therefore provide greater accuracy for toric IOL surgical planning. Notably, based on the zone mode, among all the different diameters examined, the 3-mm cornea data offered the most accurate prediction. The 3-mm zone mode is not only more representative of the actual corneal astigmatism but also it is closer to the pupillary area (24), which is actually responsible for corneal refraction, suggesting that this is the optimal option for toric IOL surgical planning in future clinical practice.

The influence of the local corneal topography on prediction accuracy should not be neglected in the eyes with the symmetric bowtie pattern of corneal astigmatism. In these eyes, the location of the steepest parts of the bowtie varies, which may lead to differences in the prediction accuracy of the different analysis modes. In particular, if the steepest part of the bowtie is located on the central cornea, the prediction accuracy is greater for the zone mode than for the ring mode at a corneal diameter of 2 mm. In contrast, if the steepest part of the bowtie is located on the peripheral cornea, the prediction accuracy is greater for the zone mode than for the ring mode at corneal diameters of 4 and 5 mm. This finding provides an insight into how to select the most appropriate analytical mode for planning toric IOL implantation according to the local corneal topography.

Some issues need to be addressed with regard to this study. The implantation of toric lenses represents a recent frontier of refractive surgery and cataract surgery, but there are limitations that cannot be addressed, even with sophisticated instruments like Pentacam. Eyes that have undergone previous operations or suffer corneal pathologies pose a great risk of surgical failure. Therefore, despite advanced instruments and more accurate analytical modes, previous incisions or scars must also be considered. For instance, cataract surgery on eyes that have undergone previous radial keratotomy poses higher risks of dehiscence and higher residual astigmatism, whereas the stabilizing suture of the radial keratotomy incision can reduce the risk of dehiscence and post-operative astigmatism (25). However, eyes with previous surgery or corneal pathologies were excluded from this study to avoid their effect on the prediction error. Furthermore, gender should always be considered in research. Studies have shown a between-sex difference in corneal astigmatism (26). In this study, no significant difference was detected in corneal astigmatism between male and female patients (*P* > 0.05), though 61% of the patients were females. Gender may be a secondary factor. A future study with a larger sample size is needed to further investigate the effect of gender on the prediction accuracy for corneal astigmatism.

In conclusion, for toric IOL surgical planning, the corneal apex may be a better reference center than the pupil center, and at a corneal diameter of ≥4 mm, the zone mode may be more accurate than the ring mode. The influence of the local corneal topography on the prediction accuracy in symmetric bowtie corneal astigmatism shall also not be neglected.

## Data Availability Statement

The raw data supporting the conclusions of this article will be made available by the authors, without undue reservation.

## Ethics Statement

The studies involving human participants were reviewed and approved by the Institutional Review Board of the Eye and Ear, Nose, and Throat Hospital of Fudan University, Shanghai, China. The patients/participants provided their written informed consent to participate in this study.

## Author Contributions

Conception and design by XZ. Analysis and interpretation of data by JM and JY. Data collection by JM, WH, and LW. Manuscript preparation by XZ, JM, JY, WH, LW, and YL. All authors contributed to the article and approved the submitted version.

## Funding

Publication of this article was supported by research grants from the National Natural Science Foundation of the People's Republic of China (Grant Nos. 81870642 and 81970780), the Clinical Research Plan of SHDC (No. SHDC2020CR4078), the Shanghai Talent Development Fund (Grant No. 201604, Shanghai, China), and the Outstanding Youth Medical Talents Program of Shanghai Health and Family Planning Commission (Grant No. 2017YQ011). The sponsor or funding organization had no role in the design or conduct of this research.

## Conflict of Interest

The authors declare that the research was conducted in the absence of any commercial or financial relationships that could be construed as a potential conflict of interest.

## Publisher's Note

All claims expressed in this article are solely those of the authors and do not necessarily represent those of their affiliated organizations, or those of the publisher, the editors and the reviewers. Any product that may be evaluated in this article, or claim that may be made by its manufacturer, is not guaranteed or endorsed by the publisher.
